# Platelet releasates promote the proliferation of hepatocellular carcinoma cells by suppressing the expression of KLF6

**DOI:** 10.1038/s41598-017-02801-1

**Published:** 2017-06-21

**Authors:** Ao-Di He, Wen Xie, Wei Song, Yuan-Yuan Ma, Gang Liu, Ming-Lu Liang, Xing-Wen Da, Guang-Qiang Yao, Bi-xiang Zhang, Cun-Ji Gao, Ji-zhou Xiang, Zhang-Yin Ming

**Affiliations:** 1 Department of Pharmacology, School of Basic Medicine, Tongji Medical College of Huazhong University of Science & Technology, 13 Hangkong Road, Wuhan, 430030 China; 2 The Key Laboratory for Drug Target Researches and Pharmacodynamic Evaluation of Hubei Province, Wuhan, 430030 China; 3 Hepatic Surgery Center, Tongji Hospital, Tongji Medical College, Huazhong University of Science and Technology, Wuhan, 430030 Hubei China; 4 Chronic Disease Research Institute, Department of Nutrition and Food Hygiene, Zhejiang University School of Public Health, Hangzhou, China

## Abstract

Platelets in the primary tumor microenvironment play crucial roles in the regulation of tumor progression, but the mechanisms underlying are poorly understood. Here, we report that platelet releasates exerted a proliferative effect on hepatocellular carcinoma (HCC) cells both *in vitro* and *in vivo*. This effect depended on a reduction of KLF6 expression in HCC cells. After incubation with either platelets or platelet granule contents, SMMC.7721 and HepG2 cells exhibited significant increases in proliferation and decreases in apoptosis. However, no effect was observed when incubating cancer cells with resuspended activated platelet pellet which exhausted of releasates. Platelet releasates also increased the population of HCC cells in the S and G2/M phases of the cell cycle and reduced the cell population in the G0/G1 phase. Moreover, knocking down KLF6 expression significantly diminished the platelet-mediated enhancement of HCC growth. In addition, blocking TGF-β signaling with the TGF-β receptor inhibitor SB431542 counteracted the effect of platelets on KLF6 expression and proliferation of HCC cells. Based on these findings, we conclude that platelet releasates, especially TGF-β, promote the proliferation of SMMC.7721 and HepG2 cells by decreasing expression of KLF6. This discovery identifies a potential new therapeutic target for the prevention and treatment of hepatocellular carcinoma.

## Introduction

Primary liver cancer is the sixth most common cancer and the second most common cause of cancer mortality worldwide^1, 2^. Unfortunately, the incidence of hepatocellular carcinoma (HCC) has been on the rise globally, especially in Asia^3^. Although new therapeutic strategies have significantly improved the survival of patients diagnosed during the early stages of this disease, the majority of patients continue to be diagnosed at an advanced stage, and their prognosis remains poor^4^. Therefore, more studies should be conducted to investigate mechanisms that drive HCC carcinogenesis.

The association between platelets and tumor cells has been recognized for centuries, starting with the introduction of Trousseau syndrome in 1865^5, 6^. Over the last 20 years, an increasing body of evidence has supported a critical role for platelet activation and the coagulation system in the progression of cancer^7–10^. Specifically, clinical studies have shown that platelets are involved in the extra-hepatic metastasis of HCC^11^, and platelet extracts have been shown to induce the growth and migration of HCC *in vitro* ^12^. However, the molecular mechanism underlying this association has not been fully explored.

Krüppel-like factor 6 (KLF6), which was initially identified as the Core Promoter Binding Protein(CPBP), is involved in the regulation of the pregnancy-specific glycoprotein genes^13^. Specifically KLF6 is a ubiquitously expressed zinc finger transcription factor that is part of a growing KLF family, which is broadly involved in growth-related signal transduction, cell proliferation, apoptosis, development, as well as angiogenesis^14, 15^. The KLF6 mRNA is widely distributed and highly enriched in the placenta both at term and during embryogenesis^16, 17^. Currently, KLF6 is characterized as a tumor suppressor gene in several human cancers^18–20^, including prostate, gastric, colon and astrocytic gliomas, and hepatocellular carcinoma^21, 22^. A recent study reported that KLF6 interacted with Sp1 to repress HCC proliferation and metastasis by regulating basigin-2 expression^23^. Furthermore, KLF6 suppresses tumor growth by regulating cell cycle and apoptosis in several cell lines^24^. Therefore, we hypothesized that the effects of platelets on tumor cells are attributable to the ability of decreasing tumor cell expression of KLF6.

Transforming growth factor beta (TGF-β) controls the proliferation and differentiation of many types of non-malignant cells and is necessary for tumor cell extravasation and metastasis^25^, and research has shown that platelets are an important source of bioavailable TGF-β for tumor cells in the circulation and at the site of extravasation^26^. Specifically, platelet derived TGF-β enhances the epithelial-mesenchymal-like transition (EMT) via the TGF-β/Smad and NF-kB pathways^26^ and increases the proliferation of ovarian cancer cells^27^. Moreover, TGF-β cooperates with KLF6 and Sp1 (specificity protein 1) to regulate target genes in cells^28^. Collectively, these observations indicate that TGF-β may play a role in the interaction between platelets and cancer cell.

Our study demonstrated that platelet granule contents released upon platelet activation promoted HCC cells growth both *in vitro* and *in vivo*, which correlated with a reduction in KLF6 expression in HCC cells. Moreover, knocking down KLF6 expression with shRNA substantially attenuated the pro-proliferative effect of platelets, whereas blocking TGF-β signaling with a TGF-β receptor inhibitor abrogated the stimulatory effect of platelets on HCC cells. Together, these findings suggest that the down-regulation of KLF6 expression by platelet-derived TGF-β plays an essential role in the pro-proliferative effect of platelets on HCC cells.

## Results

### Treatment of tumor cells with platelet releasates controls proliferation, cell cycle and apoptosis

**Figure 1:**
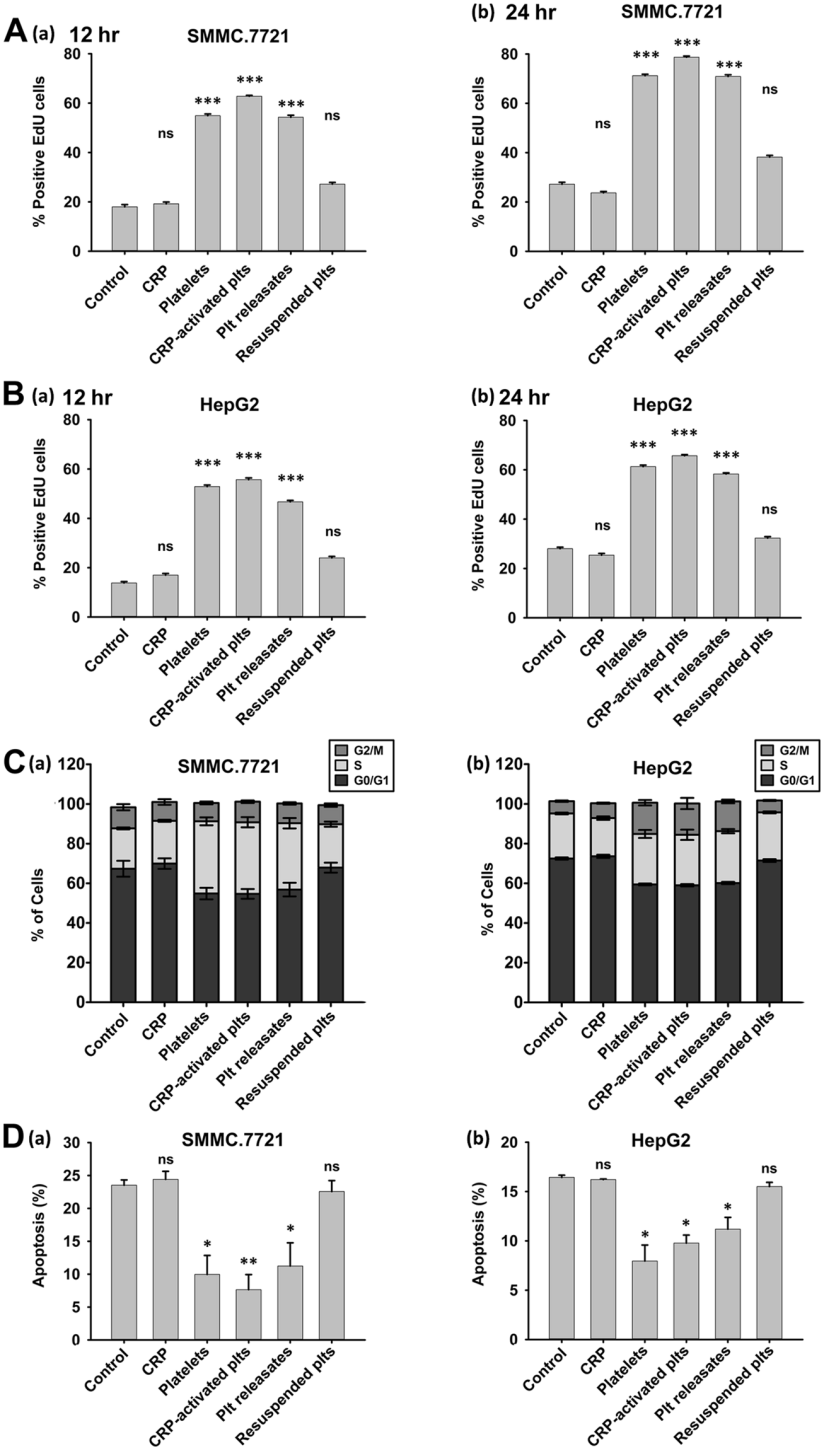
Platelets mediate the proliferation, cell cycle and apoptosis of HCC cells *in vitro*. (**A,B**) SMMC.7721 (**A**) and HepG2 (**B**) cells were incubated with resting platelets, platelets treated with specific stimulator CRP (0.8 μg/ml), releasates of CRP-activated platelets, or the resuspended CRP-activated platelets pellet. The proliferation rate of HCC cells was assessed by measuring EdU incorporation after 12 (a) or 24 (b) hours of the above-indicated treatment, and cells cultured in fresh medium served as a control. EdU-positive cells were quantified in 5 randomly selected fields at x200 magnification, and the experiment was repeated 3 times. The results were expressed as the mean ± SEM (***p < 0.001 compared with the control). (**C**) Cell cycle analysis of SMMC.7721 (a) and HepG2 (b) cells after treatment with platelets, their releasates or resuspended platelets pellets for 24 hours (n = 3). (**D**) Apoptosis analysis of SMMC.7721 (a) and HepG2 (b) cells after treatment with platelets, their releasates or resuspended platelets pellets for 24 hours (n = 3), and cells cultured in fresh medium served as a control. *p < 0.05 and **p < 0.01 were determined by Student’s t-test, plts: platelets.

To establish the role of platelet and its secretions in HCC cell proliferation, we incubated HCC cells with the following platelets for 12 or 24 hours: unstimulated platelets, CRP-activated platelets, supernatants of CRP-activated platelets (which served as platelet releasates), and resuspended activated platelets in fresh DMEM (activated platelet pellets without any releasates). Treatment with resting platelets and CRP-activated platelets resulted in a significant increase in SMMC.7721 proliferation compared with that in the control group (Fig. 1A(a) and (b)). Supernatants from activated platelets also promoted tumor cell growth, whereas treatment with resuspended platelets had less influence on SMMC.7721 proliferation (Fig. 1A(a) and (b)). We observed similar results in HepG2 cells (Fig. 1B). To better understand the mechanisms underlying the platelet-mediated accelerated growth of HCC cells, the cell cycle and apoptosis were analyzed. Compared with the control group, SMMC.7721 and HepG2 cells co-cultured with platelets and their releasates exhibited decreases in the percentage of cells in the G0/G1 phase and increases in the percentages of cells in the S and G2/M phases, whereas these percentages did not significantly differ between the resuspended platelet treatment and control groups (Fig. 1C). Moreover, an analysis of FITC-Annexin V^+^ tumor cells (Fig. 1D and Supplemental Fig. 1) showed reduced apoptosis after treatment with platelets or their releasates for 24 hours but not after treatment with resuspended platelets. Therefore, direct contact between platelets and HCC cells was not necessary for the proliferation response.

### Platelet releasates reduce the expression of KLF6 in hepatocellular carcinoma cells

**Figure 2:**
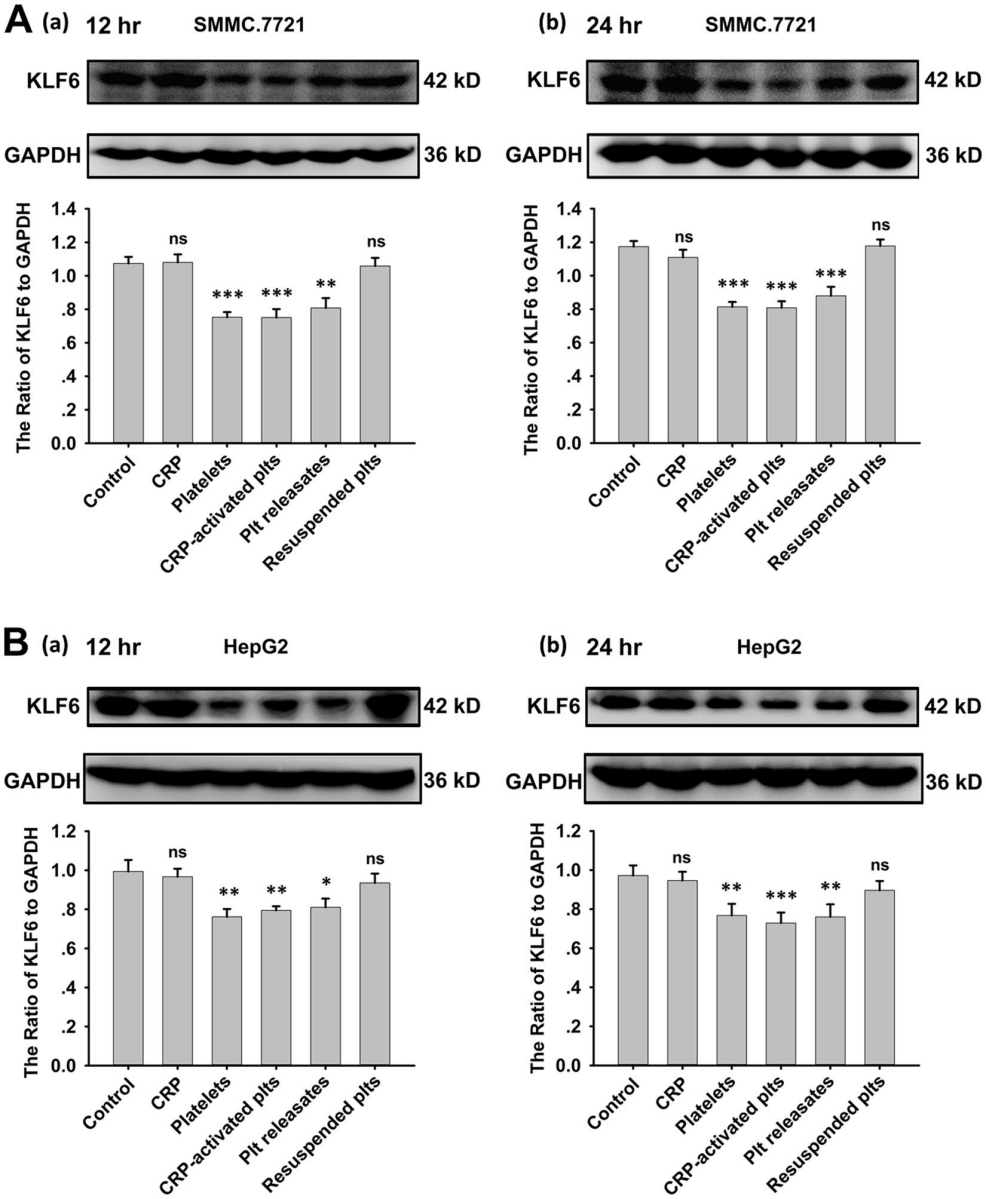
Expression of KLF6 in HCC cells was down-regulated by platelets. (**A,B**) After incubation with resting platelets, CRP-activated platelets, platelets releasates, and the resuspended platelets pellets for 12 (a) or 24 (b) hours, KLF6 expression in SMMC.7721 (**A**) and HepG2 (**B**) cells was measured by a Western blot analysis. A quantitative analysis of the ratio of KLF6 to GAPDH expression is shown, and data are presented as the mean ± SEM (n = 9 for SMMC.7721, and n = 8 for HepG2, **p < 0.01 and ***p < 0.001 compared with control).

Given the evidences that KLF6 has been identified as a tumor suppressor gene and regulates genes controlling the cell cycle, apoptosis and differentiation^29^, we investigated the role of KLF6 in the interaction between platelets and tumor cells. To this end, the expression levels of KLF6 in HCC cells and platelets were evaluated. Both SMMC.7721 and HepG2 cells expressed KLF6, which was deficient in platelets (Supplemental Fig. 2). After SMMC.7721 and HepG2cells were incubated with resting or CRP-stimulated platelets for 12 and 24 hours, the expression of KLF6 in both groups significantly decreased compared with the control group, respectively (Fig. 2A and B). The supernatants also suppressed KLF6 expression in HCC cells. Conversely, platelet pellets resuspended in fresh medium did not significantly influence KLF6 expression (Fig. 2A and B).

### Loss of KLF6 blocks platelet-mediated tumor cell proliferation and cell cycle progression

**Figure 3:**
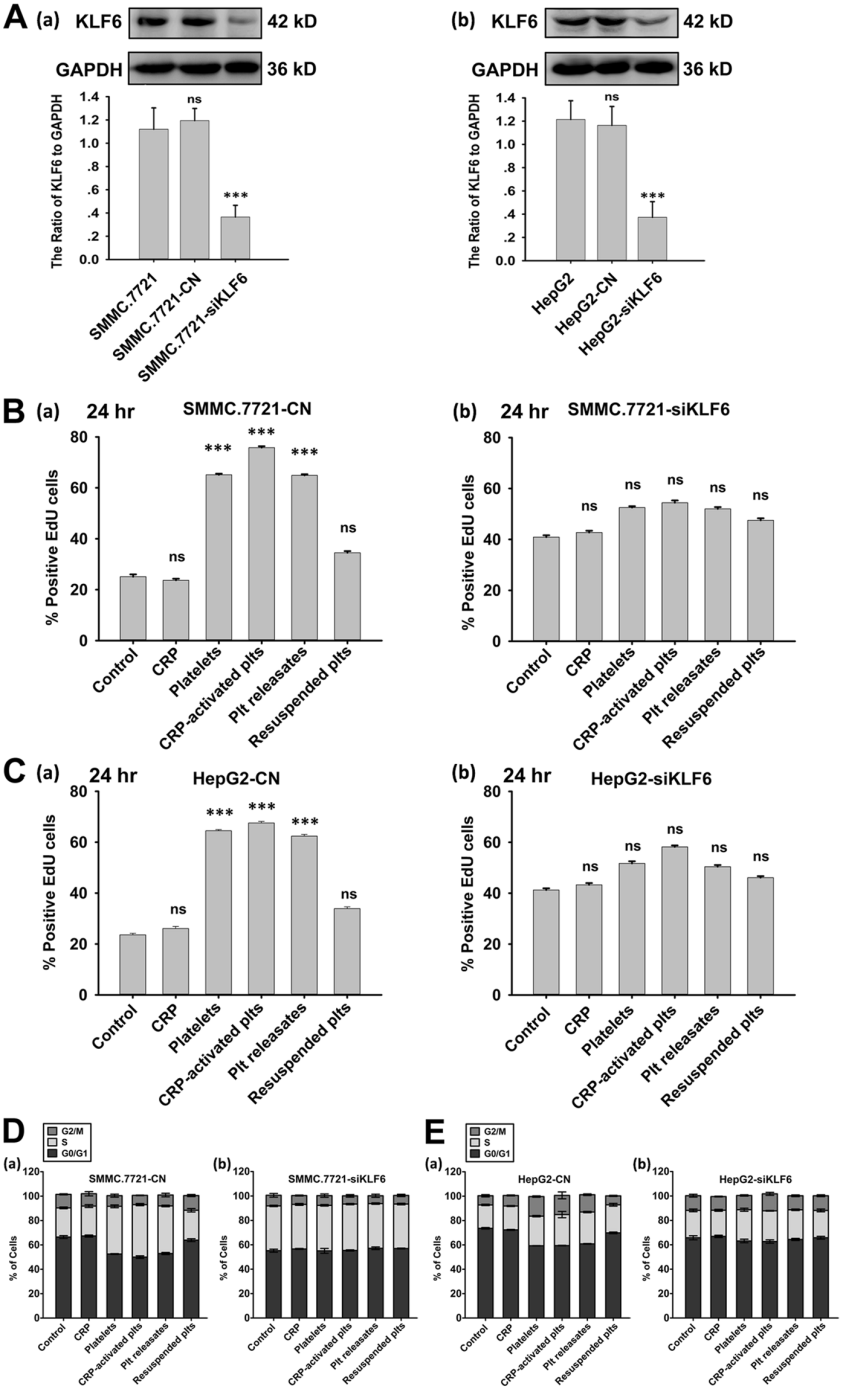
Knocking down KLF6 expression in HCC cells abrogated the proliferative effect of platelets. (**A**) SMMC.7721 (a) and HepG2 (b) cells were transfected with either control shRNA (CN) or KLF6 shRNA (siKLF6) for 96 hours, and KLF6 expression was visualized by a Western blot. GAPDH served as a loading control. A quantitative analysis of the ratio of KLF6 to GAPDH expression is shown, and data are presented as the mean ± SEM (n = 3, ***p < 0.001 compared with control). (**B,C**) SMMC.7721 (**B**) and HepG2 (**C**) cells stably transfected with control shRNA (a, CN) or KLF6 shRNA (b, siKLF6) were incubated with the indicated components. EdU incorporation was measured to determine HCC cell proliferation after incubation for 24 hours, and the respective cell type cultured in fresh medium served as a control. The data are presented as the mean ± SEM (***p < 0.001 compared with controls. Five randomly selected fields were examined at x200 magnification, and the experiment was repeated three times). (**D,E**) Cell cycle analysis of SMMC.7721 cells transfected with control shRNA (**D**(a)), SMMC.7721 cells transfected with KLF6 shRNA (**D**(b)), HepG2 cells transfected with control shRNA (**E**(a)), and HepG2 cells transfected with control shRNA (**E**(b)).

To gain better insight into the critical role of KLF6 in the platelet-mediated pro-proliferative effects on HCC cells, we transfected SMMC.7721 and HepG2 cells with KLF6 shRNA to knockdown its expression (Fig. 3A). Compared with scrambled shRNA-transfected SMMC.7721 cells, KLF6 shRNA-transfected cells exhibited an improved growth rate, and this improvement correlated with the reduction in KLF6 expression (Fig. 3B(b)). After 12 or 24 hours of treatment with platelets and their releasates, a significant increase in the proliferation of control shRNA-transfected SMMC.7721 cells was observed compared to cultures grown in the absence of platelets or the presence of resuspended platelets. However, reducing KLF6 expressionin SMMC.7721 cells abrogated the tumor cell growth induced by platelets and their releasates (Fig. 3B and Supplemental Fig. 3A). Similar results were also observed in HepG2 cells (Fig. 3C and Supplemental Fig. 3B).

Studies have suggested that KLF6 is related to cell cycle progression in several carcinoma cell lines^24^. Therefore, we examined the involvement of KLF6 in the platelet-mediated cell cycle progression of HCC cells. As shown in Fig. 3D(a) and E(a), platelets and their secretions, but not resuspended platelets, modestly reduced the cell population in the G0/G1 phase and increased the cell populations in the S and G2/M phases for scrambled shRNA-transfected HCC cells. In contrast, both SMMC.7721 and HepG2 cells transfected with KLF6 shRNA exhibited higher percentage of cells in the G0/G1 phase and lower percentages of cells in the S and G2/M phases compared with control shRNA-transfected cells (Fig. 3D(b) and E(b)). Treatment with neither platelets nor releasates regulated cell cycle progression in KLF6-silenced HCC cells (Fig. 3D,E), indicating that platelets mediated HCC cell growth and cell cycle progression via KLF6.

### Platelet releasates prompt ***in vivo*** tumor growth in nude mice

**Figure 4:**
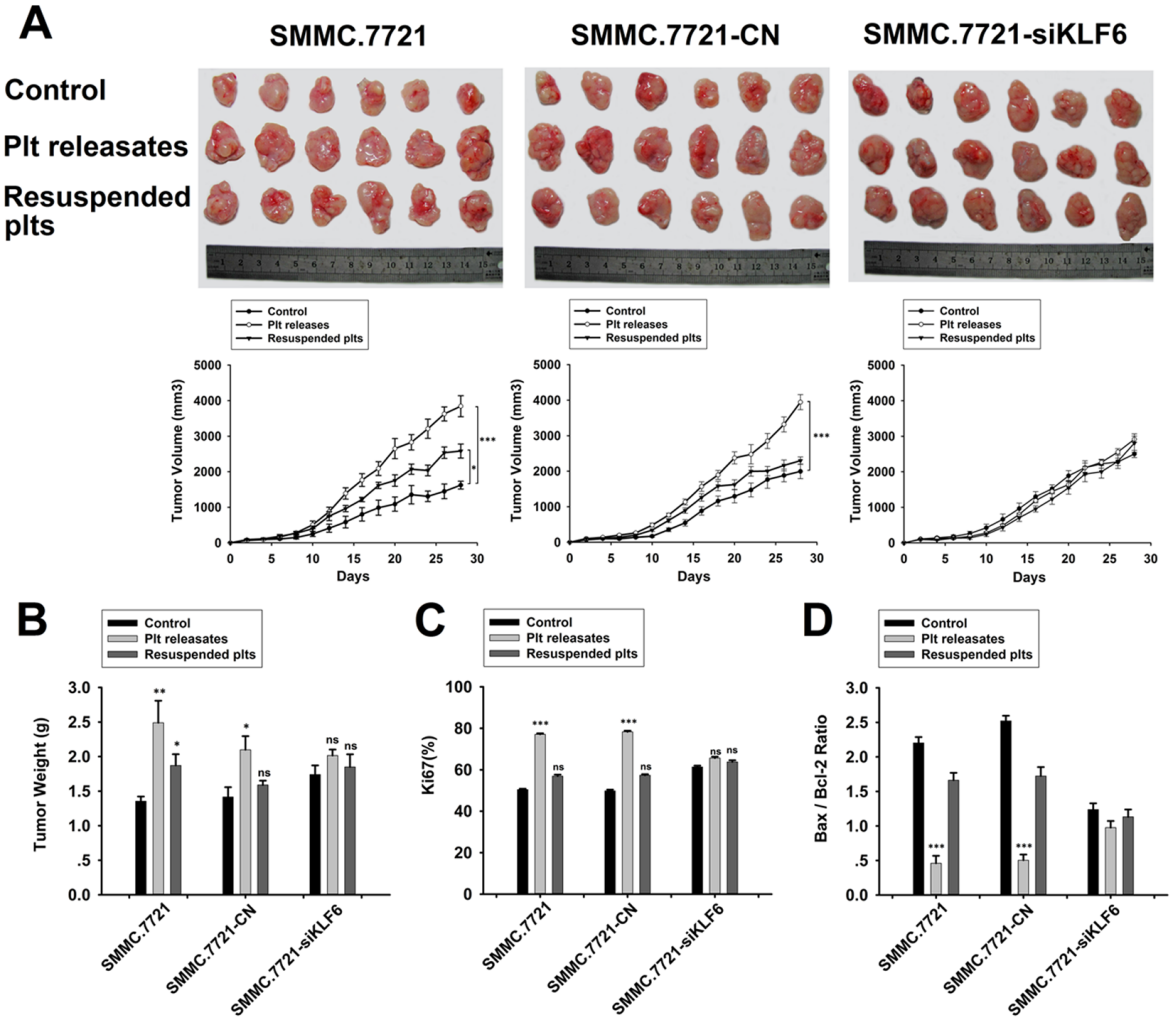
Effect of platelets on SMMC.7721 tumor growth *in vivo*. (**A**) SMMC.7721 cells and control or siKLF6-transfected SMMC.7721 cells were treated with platelet releasate and resuspended platelet pellets for 24 hours, and these tumor cells were then subcutaneously injected into the flanks of nude mice. Representative images (upper panel) of tumors and statistical analyses of tumor volume data (lower panel) from each group are shown. ***p < 0.001 and *p < 0.05 compared with controls. (**B**) The tumor weights of SC tumors are shown. **p < 0.01 and *p < 0.05 compared with controls. (**C**) The quantification of Ki67 in tumors from each group is shown. Positive cells were quantified in 5 randomly selected fields at x200 magnification. (**D**) Bax and Bcl-2 were quantified in tumors of each group, and the ratio of Bax to Bcl-2 is shown. ***p < 0.001 compared with controls.

Next, we investigated the effect of platelet releasates on tumor growth *in vivo* using SMMC.7721 established subcutaneously tumors. After tumor cells had been treated with platelet releasates, the mice injected with SMMC.7721 cells or scrambled shRNA-transfected SMMC.7721 cells exhibited larger (Fig. 4A) and heavier (Fig. 4B) tumors than mice in the control group, whereas no significant differences were observed after SMMC.7721 cells were treated with resuspended platelets. Conversely, even KLF6-silenced SMMC.7721 tumors were larger than scrambled shRNA-transfected SMMC.7721 tumors, they were not affected by treatment with platelet releasates or resuspended platelets (Fig. 4A and B). Additionally, both SMMC.7721 and scrambled shRNA-transfected SMMC.7721 tumors resected from platelet releasates-treated group exhibited significantly higher proliferation (Fig. 4C and Supplemental Fig. 4) and a lower apoptosis rate (Fig. 4D) compared with tumors excised from control mice, as based on the numbers of Ki67-positive tumor cells and the ratio of the relative intensities of Bax and Bcl-2 in tumor cells. These differences were abolished in the KLF6-silenced group. Thus, KLF6 is likely the main factor responsible for platelet releasates-mediated tumor growth.

### Platelet derived TGF-β mediates KLF6 expression and induces the proliferation of HCC cells ***in vitro***

**Figure 5:**
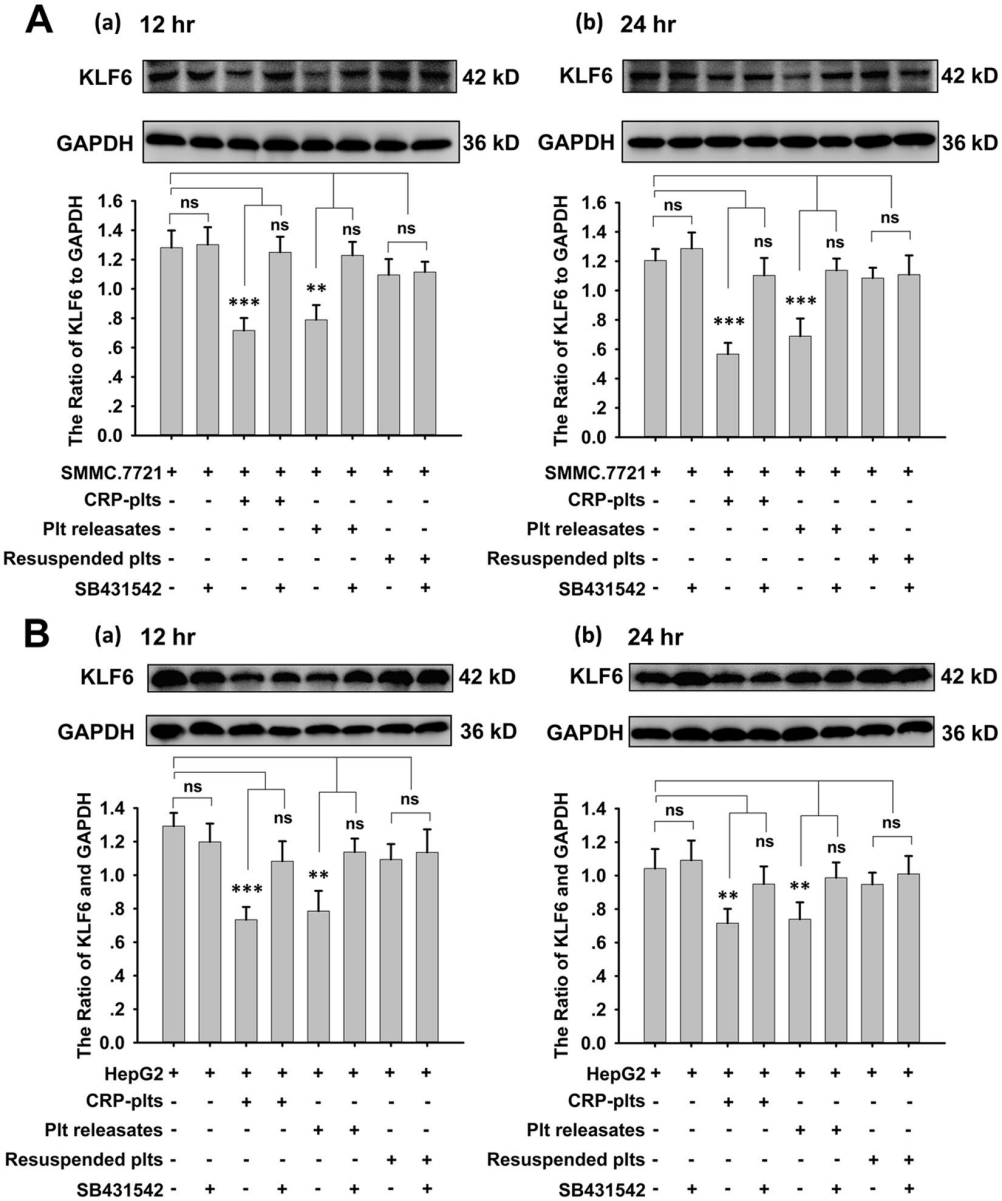
Platelet-derived TGF-β regulates the expression of KLF6 in HCC cells. SMMC.7721 (**A**) and HepG2 (**B**) cells were incubated with CRP-activated platelets, platelets releasates, and resuspended platelet pellets in the absence and presence of TGF-β receptor inhibitor SB431542 (10 μM). KLF6 expression was detected by a Western blot analysis in HCC cells after 12 (a) or 24 (b) hours of treatment. Data were quantified and are shown as the mean ± SEM (n = 5, **p < 0.01 compared with controls).

**Figure 6:**
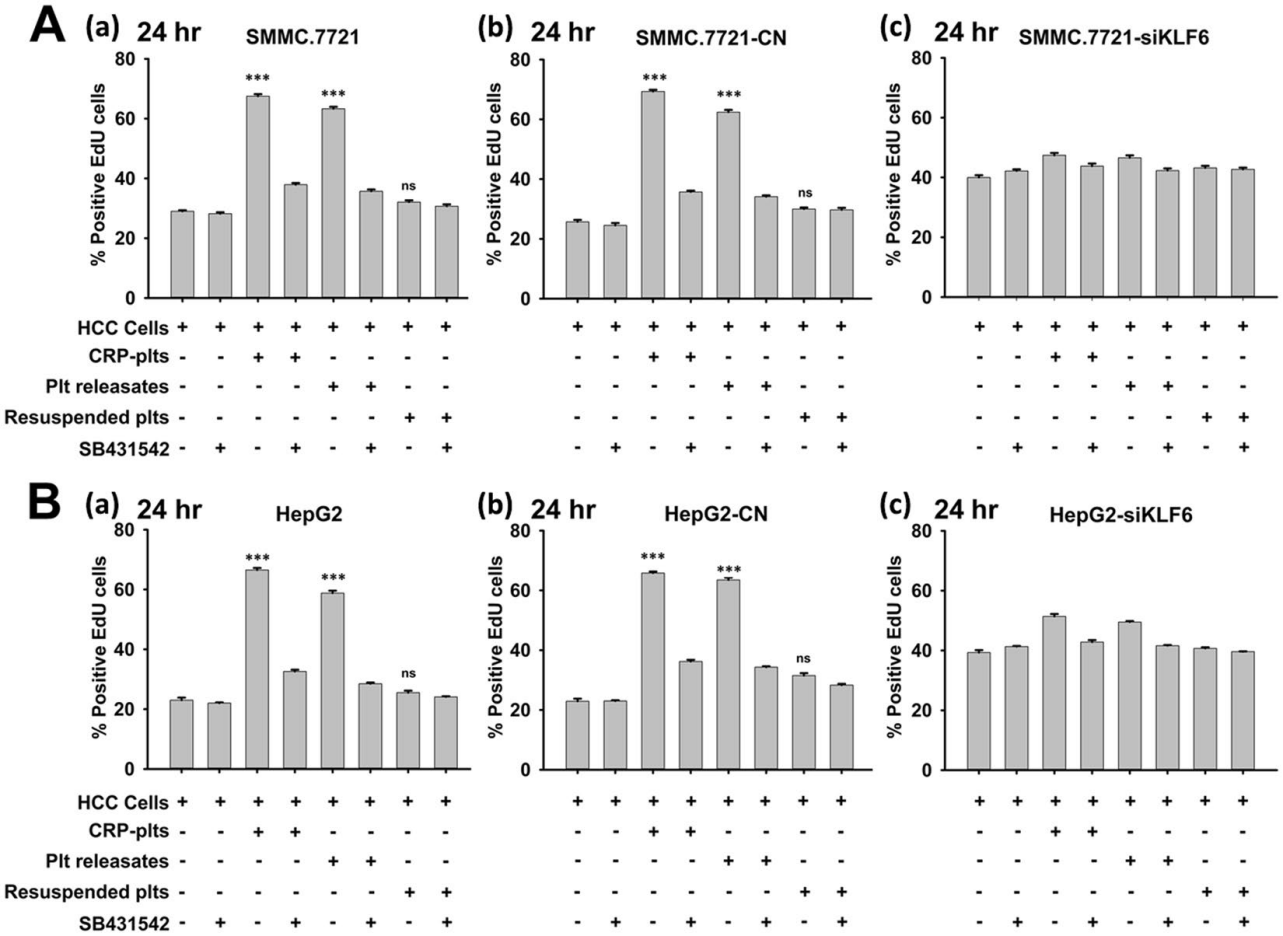
Platelet-derived TGF-β promotes HCC cell proliferation. (**A**) SMMC.7721 (a), control shRNA-transfected SMMC.7721 (b) and KLF6-silenced SMMC.7721 (c) cells were treated with platelets and their releasates in the absence and presence of TGF-β receptor inhibitor SB431542 (10 μM). The EdU incorporation assay was used to determine the effects of platelets on tumor cell proliferationafter24 hours, and cells cultured in fresh medium were used as a control. (**B**) Quantification of EdU-positive HepG2 (a), control shRNA-transfected HepG2 (b) and KLF6-silenced HepG2 (c) cells, which had been treated with platelets and their releasates in the absence and presence of TGF-β receptor inhibitor SB431542 (10 μM) for 24 hours; cells cultured in fresh medium were used as a control. For (**A**) and (**B**), each experiment was repeated at least 3 times in triplicates. Five fields were randomly selected and examined at x200 magnification for each group. Data are presented as the mean ± SEM. (***p < 0.001 compared with controls).

In our earlier observations, platelet releasates enhanced the proliferation of HCC cells. Because most of the TGF-β in the serum is contained in platelet α-granules^30^ and recombinant TGF-β1 could reduce expression of KLF6 in HCC cells (Supplemental Fig. 5), we hypothesized that platelet-derived TGF-β is involved in tumor growth and affects KLF6 expression. To investigate this hypothesis, HCC cells were pre-incubated with SB431542, a TGF-β receptor blocker, and treated with platelets or their releasate. Both CRP (0.8 μg/ml)-treated platelets and their releasate reduced the expression of KLF6 in HCC cells at the 12 and 24 hour time points, and these effects could be blocked by SB431542 (10 μM) (Fig. 5A and B). However, KLF6 expression in HCC cells was not altered after co-incubation with resuspended platelets, irrespective of the presence of SB431542 (Fig. 5A and B). Consistent with these results, SB431542 attenuated platelet- and releasate-mediated HCC cell proliferation, irrespective of treatment duration 12 hours (Supplemental Fig. 6A(a) and B(a)) or 24 hours (Fig. 6A(a) and B(a)). Cell growth did not significantly differ between the resuspended platelet-treated groups and the respective controls. In addition, this inhibition effect of SB431542 was abolished in both KLF6-silenced SMMC.7721 and KLF6-silenced HepG2 cells (Fig. 6A(c) and B(c) and Supplemental Fig. 6A(c), B(c)). Taken together, these results show that platelet-derived TGF-β is necessary to reduce the expression of KLF6 and promote the proliferation of HCC cells.

## Discussion

**Figure 7:**
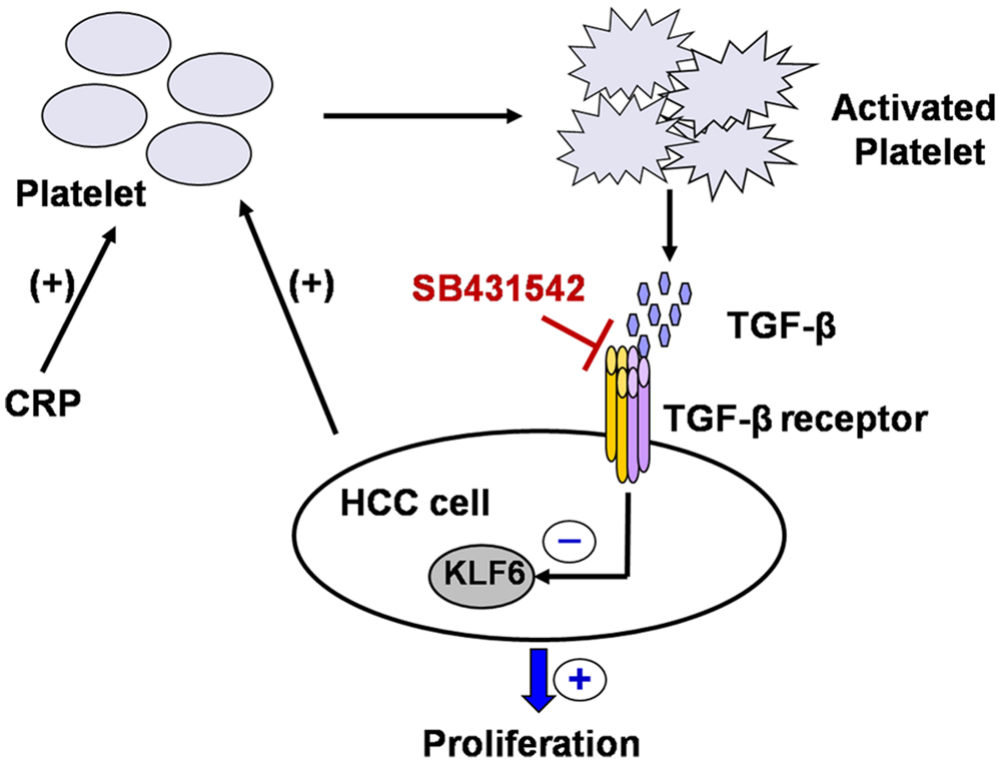
Schematic representation of KLF6-dependent, platelet-induced tumor cell proliferation. The platelet-induced proliferation of HCC cells requires platelet by the platelet-specific agonist CRP or HCC cells. Activated platelets then secrete granules containing cytokines, such as TGF-β, that promote tumor cell growth and mediate the cell cycle and apoptosis of tumor cells by down-regulation of KLF6 expression.

Signals provided by the primary tumor microenvironment are vital modulators of tumor cell progression^31^. Specifically, platelets have been recognized as key players in the regulation of tumor metastasis and angiogenesis^32, 33^. In support of this concept, we found that washed human platelets actively signal to HCC cells to promote their proliferation both *in vitro* and *in vivo*. This effect is most dependent on the secretions of activated platelets because resuspended activated platelets in fresh medium cannot alter tumor growth, cell cycle or apoptosis. Moreover, we found that platelet releasate reduced the expression of KLF6 in HCC cells, and TGF-β in platelet α-granules is most likely involved in this interaction (Fig. 7).

Extensive experimental evidence shows that platelets support tumor progression^34^. In our study, both CRP-activated and non-activated platelets as well as activated platelet releasate strongly increased HCC cell proliferation, indicating that platelets play an important role in liver cancer growth. However, the resuspended activated platelet pellet did not increase HCC cell proliferation, which implies that platelet releasate contributes to HCC cell growth. Our data also show that resting platelets and CRP-stimulated platelets exert a similar effect on HCC cell growth, which implying the potential of HCC cells on activating platelets. Accordingly, recent studies indicate that platelet activation is amplified in many cancers and found within the tumor vasculature^35–37^. However, the effect of HCC cells on platelet activation requires further investigation. Furthermore, co-incubation with platelets resulted in a large percentage of HCC cells in the S phase, a lower percentage of cells in theG0/G1 phase and decreased apoptosis. Clinically, tumor growth is usually accompanied by an increased tendency towards platelet abnormalities in cancer patients^33, 38^. Specifically, platelet releasate promotes HCC cell growth via the tumor vasculature, and tumor cells also could gain increased proliferation capacities after invaded into blood vessels.

In several tumor cell lines, platelets affected EMT, invasion and metastasis, and these effects are not only mediated by secretions but also require additional platelet-bound factors^26^. In contrast, the pro-proliferative effect of platelets on HCC cells depends on releasates from activated platelets and to a lesser extent on platelet pellets, which had secreted all of their granular components. In our study, tumor cell growth was enhanced by releasates obtained from purified platelets stimulated with CRP. In addition, incubation with platelet releasates, but not pellet fractions, changed the cell cycle and apoptosis of HCC cells. This finding is consistent with the close association between platelets and ovarian cancer cell proliferation as well as platelet extract and HCC cell progression^27^.

Most importantly, we show that treating HCC cells with platelets and their releasates is correlated with a reduction in KLF6 expression. Similarly, this effect of platelets on KLF6 expression was independent of direct contact between platelets and tumor cells because pellets of CRP-activated platelets did not affect the expression of KLF6 in HCC cells. KLF6 is known as a tumor suppressor gene, and its frequent down-regulation has been implicated in several human cancers. Specifically, reduced KLF6 expression is associated with decreased survival in patients with prostate^39, 40^ and lung cancers^41^. In HCC, a loss and/or mutation of KLF6 resulting in its inactivation and/or down-regulation contributes to HCC pathogenesis by affecting genes that control cell proliferation and differentiation^21, 23, 42^. Here, we found that HCC cells transfected with KLF6 shRNA exhibit increased growth both *in vitro* and *in vivo* because larger percentages of the cell population are in the S and G2/M phases and a smaller percentage of the cell population is in the G0/G1 phase. These results further confirm that KLF6 suppresses HCC proliferation. Moreover, the lower expression of KLF6 abrogated the pro-proliferative effect of platelets compared with scrambled shRNA-transfected HCC cells. The cell cycle of KLF6-silenced HCC cells also remains unchanged after treatment with platelets and their releasates. Although platelet releasates can promote the growth of SMMC.7721 cells transfected with scrambled shRNA *in vivo*, the volumes of tumors formed by KLF6-silenced SMMC.7721 cells in mice was not affected by the presence of platelet releasate. Moreover, tumors formed by both SMMC.7721 and scrambled shRNA-transfected SMMC.7721 cells in mice showed a lower apoptosis rate after treatment with platelet releasates, and these differences were abolished in the KLF6-silenced group. Taken together, these data clearly demonstrate that the pro-proliferative effect of platelets on HCC cells is mediated by KLF6 and independent of a direct interaction between platelets and tumor cells.

Reduced KLF6 expression occurs at early stages during HCC progression, which is attributable to control cell growth and apoptosis by regulation of multiple target genes, such as tumor suppressor genes (p21, E-cadherin, p53), oncogenes (Rb, β-catenin, pituitary tumor-transforming gene 1 (PTTG1), mouse double minute 2 homolog (MDM2)), pro-apoptosis gene (Bax) and anti-apoptosis gene (Bcl-xL)^19, 22, 29^. In our study, SMMC.7721 cells treated with platelet releasates for the initial 24 hours exhibited hyper-proliferation and lower-apoptosis during the *in vivo* experiment up to 28 days, owing to the down-regulation of KLF6 expression in SMMC.7721 cells by platelet releasates, which mediated its downstream effectors and leading to an increased proliferation and reduced apoptosis. Moreover, previous studies have shown that KLF6 silenced tumors expressed an increase in VEGF concentration and up-regulated angiogenesis-related genes^43^. As we known tumor angiogenesis is an essential determinant for primary and metastatic tumor growth. Therefore, effect of KLF6 *in vivo* might be also correlated with their effect on angiogenesis.

Several signaling molecules, including TGF-β, PDGF, VEGF and angiopoietin, are abundant in platelets and may therefore impact tumor cell behavior^44^. Our findings indicate the promoting effects of platelet releasates on HCC growth are in large part mediated by the TGF-β signaling pathway. Previous studies have suggested that the concentrations of TGF-β in platelets are many-fold higher than those in most cell types^30^ and that platelets are the main source of bioavailable TGF-β for tumor cells in the circulation^26^. Moreover, platelets can secrete TGF-β after stimulation with agonists and several tumor cells^26, 45^. Consistent with our hypothesis, blocking TGF-β signaling with a TGF-β receptor inhibitor abolished the platelet releasate-induced proliferation of HCC cells and down-regulated KLF6 expression. This finding is supported by the observation that TGF-β is involved in the regulation of KLF6 expression in various cells^28^. Thus, platelets promote tumor cell proliferation to potentiate a transcriptional response in tumor cells to platelet-derived TGF-β.

In conclusion, these data reveal that KLF6 plays a pivotal role in the contribution of platelet releasates to the proliferation of HCC cells. Moreover, platelets actively signal to tumor cells via TGF-β stored in α-granules. These findings broaden our knowledge about the role of platelets in tumor progression, which may offer a novel treatment strategy for hepatocellular carcinoma.

## Methods

### Cell culture and stable transfection

The human hepatoma cell lines SMMC.7721 and HepG2 were purchased from the Chinese Center for Type Culture Collection (CCTCC, Wuhan, China) and routinely cultured in Dulbecco’s modified Eagles’ medium (DMEM, HyClone, Logan, UT, US) supplemented with 10% fetal bovine serum (FBS, Gibco, Grand Island, NY). The cells were incubated in a humidified incubator containing 5% CO_2_ at 37 °C. Both cell lines were supplied with fresh medium every 24 hours and subcultured twice weekly. Lentiviruses containing shRNA against KLF6 and control non-targeting shRNA were obtained from GeneChem Co, Ltd, (Shanghai, China). HCC cells were seeded in six-well plates and transfected with concentrated lentivirus in the presence of polybrene (10 μg/ml, Sigma-Aldrich, St. Louis, MO, US) according to the manufacturer’s instructions. When green fluorescent protein (GFP) expression exceeded 80% in each group, cells were selected by using puromycin (5 μg/ml) and Western blot analysis was performed to examine the transduction efficiency. Selected cells in which KLF6 was stably knocked down were used for the following experiments.

### Treatment of tumor cells with washed human platelets

Human blood was collected from healthy and aspirin-free volunteers who had provided informed consent. Washed platelets were prepared as described previously^46, 47^. The platelets (2 × 10^8^/ml) were activated by its specific agonist collagen-related peptide (CRP, 0.8 μg/ml), which was kindly provided as a gift by Dr. Debra K. Newman at the Blood Center of Wisconsin. After being fully activated, the platelet suspensions were centrifuged at 1000 g for 10 min to obtain platelet releasates from the supernatant, and the sediments, which were consisted of activated platelets, was resuspended in fresh DMEM. For co-incubation, HCC cells were seeded in plates overnight and then treated with washed human platelets, platelet releasates or resuspended activated platelets and incubated for 12 or 24 hours at 37 °C. In some experiments, HCC cells were treated with human recombinant TGF-β1 (10 ng/ml, Sigma-Aldrich, St. Louis, MO, US) or TGF-β receptor inhibitor SB431542 (10 μM Selleckchem, TX, US) before incubation with human platelets.

All experimental procedures were approved by the Ethics Committee for the Use of Human Subjects of Huazhong University of Science and Technology. These studies were conducted in accordance with the Declaration of Helsinki.

### Cell proliferation assay

HCC cells were seeded into 24-well plates at a density of 2,000 cells/well and incubated overnight before being treated with platelets as indicated. Proliferation was assessed based on the incorporation of fluorescence-conjugated EdU (5-ethynil-2′-deoxyuridine) into newly synthesized DNA according to the manufacturer’s instructions (Click-iT^®^ Plus EdU Imaging Kits, Invitrogen, Life Technologies, CA, US). Cells were imaged with fluorescence microscopy, and EdU-positive cells were quantified in 5 randomly selected fields at x200 magnification.

### Cell cycle assay

HCC cells were seeded in 6-well plates at a density of 10^5^ cells/well and incubated overnight at 37 °C. Platelets were added, and the cells were further incubated for 12 or 24 hours. The cells were then trypsinized and fixed in 70% ethanol overnight at 4 °C. Propidium iodide (PI) containing RNase (BD Biosciences, San Jose, CA, US) was used to stain the DNA at room temperature for 15 min. Flow cytometry was performed using a BD FACS Calibur flow cytometer, and cell cycle distributions were calculated using the FlowJo software.

### Apoptosis assay

HCC cells were co-incubated with platelets as described previously. The FITC AnnexinV Apoptosis Detection Kit I (BD Biosciences, San Jose, CA, US) was used according to the manufacturer’s protocol. After being stained with FITC Annexin V and PI, the cells were analyzed by flow cytometry, and the percentage of FITC Annexin V-positive cells was evaluated.

### Western blot

The cells were lysed on ice by scraping them in lysis buffer supplemented with 2% protease inhibitors (Calbiochem, MA, US). The proteins were separated by SDS-PAGE and transferred to PVDF membranes. The membranes were incubated with a primary mouse antibody against KLF6 (1:250, Santa Cruz Biotechnology, CA, US), followed by incubation with a secondary antibody. The resultant signals were detected using enhanced chemiluminescence (Thermo Scientific, USA).

### Tumor models

Male BALB/c (nu/nu) mice aged 4–6 weeks were housed under specific pathogen-free (SPF) conditions and cared for according to the institutional guidelines for animal care. For *in vivo* tumorigenicity assay, SMMC.7721 and shRNA-transfected SMMC.7721 tumor cells were treated with platelet releasates or resuspended platelets for 24 hours. Then 2 × 10^6^ tumor cells in 100 μl PBS were subcutaneously injected into the flanks of nude mice. Six mice from each group were monitored once every 2 days, and tumor length and width were measured. All mice were sacrificed 4 weeks later, and the tumors were weighed.

All of the animal studies met the National Institutes of Health (NIH) guidelines and were approved by the Committee on the Ethics of Animal Experiments of the Tongji Medical College, HUST (No. 00139724).

### Immunohistochemistry

The tumor tissues were dissected, fixed in 4% paraformaldehyde overnight and embedded in paraffin before being sectioned. Proliferation and apoptosis were assessed using anti-Ki67, anti-Bax, and anti-Bcl-2 antibodies. The numbers of Ki67-, Bax- and Bcl-2-positive cells were quantified in 5 randomly selected fields at x200 magnification.

### Statistical analysis

All data are presented as the mean ± SEM. Differences between groups were evaluated using a 2-tailed Student’s *t* test or one-way ANOVA, adjusting for multiple comparisons. For all statistical analyses, *P* < 0.05 was considered significant.

### Data Availability

All data generated or analysed during this study are included in this published article and its Supplementary Information files.

## Electronic supplementary material

Supplementary Information
