# Supplementary material for: Platelet releasates promote the proliferation of hepatocellular carcinoma cells by suppressing the expression of KLF6
Source: Sci Rep. 2017 Jun 21;7:3989. doi: 10.1038/s41598-017-02801-1 (PMC5479859; doi:10.1038/s41598-017-02801-1)
Supplement: Supplementary file 1 — Supplementary Information [file 41598_2017_2801_MOESM1_ESM.doc]

**Supplementary Information**

**Platelet releasates** **promote the proliferation of** **hepatocellular carcinoma cells** **by suppressing the expression of KLF6**

**Ao-Di He^1^, Wen Xie^1^, Wei Song^1^, Yuan-Yuan Ma^1^, Gang Liu^1^, Ming-Lu Liang^1^, Xing-Wen Da^1^, Guang-Qiang Yao^1^,** **Bi-xiang Zhang^3^, Cun-Ji Gao^4^, Ji-zhou Xiang^1^, Zhang-Yin Ming^1,2^***

1. Department of Pharmacology, School of Basic Medicine, Tongji Medical College of Huazhong University of Science & Technology, 13Hangkong Road, Wuhan 430030, China

2. The Key Laboratory for Drug Target Researches and Pharmacodynamic Evaluation of Hubei Province, Wuhan 430030, China

3. Hepatic Surgery Center, Tongji Hospital, Tongji Medical College, Huazhong University of Science and Technology, Wuhan430030, Hubei, China

4. Chronic Disease Research Institute, Department of Nutrition and Food Hygiene, Zhejiang University School of Public Health, Hangzhou, China

*Corresponding author: Zhang-Yin Ming M.D. Ph.D.

Department of Pharmacology, School of Basic Medicine, Tongji Medical College of Huazhong University of Science & Technology, Wuhan, China.

Tel.: + 86 27 83691761; Fax: + 86 27 83692608.E-mail: [zyming@hust.edu.cn](mailto:zyming@hust.edu.cn).

**
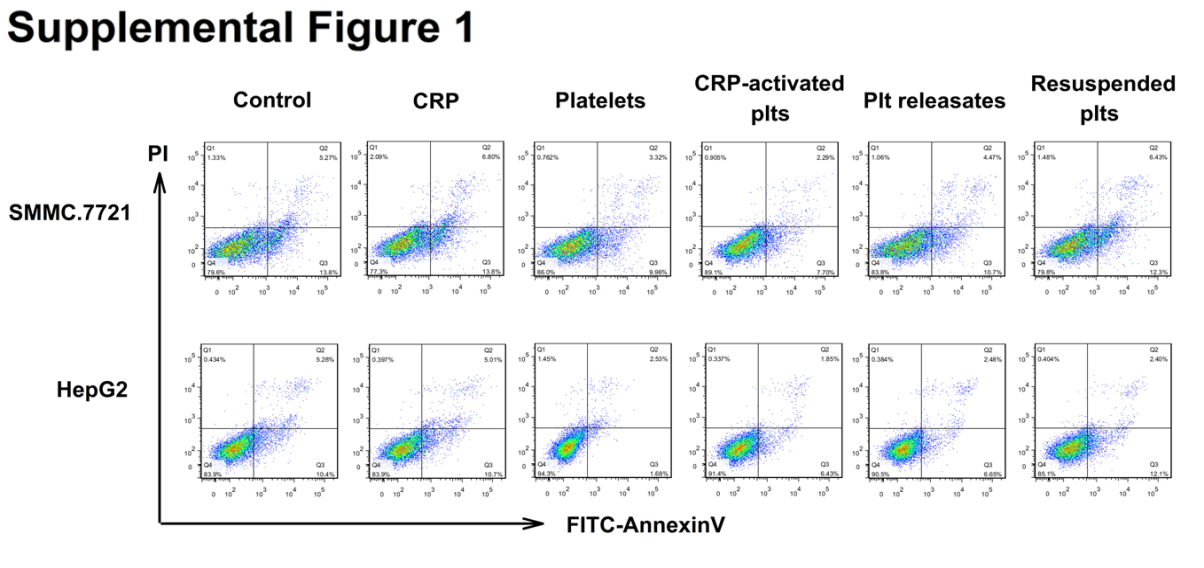
**

**Supplemental Figure 1. Platelets and their releasates regulate the apoptosis of HCC cells.** The apoptosis of SMMC.7721 and HepG2 cells was analyzed after treatment with platelets (plts), platelet releasates and resuspended platelets. Images representative of the expression of PI (Propidium Iodide) and FITC-AnnexinV in HCC cells are shown.

**
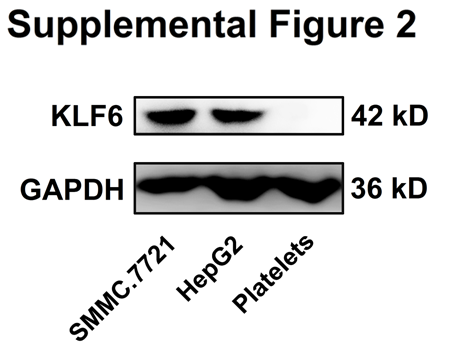
**

**Supplemental Figure 2. Expression of KLF6 in HCC cells and platelets.** Western blot analysis of KLF6 expression in SMMC.7721 cells, HepG2 cells and washed platelets. GAPDH served as a loading control.

**
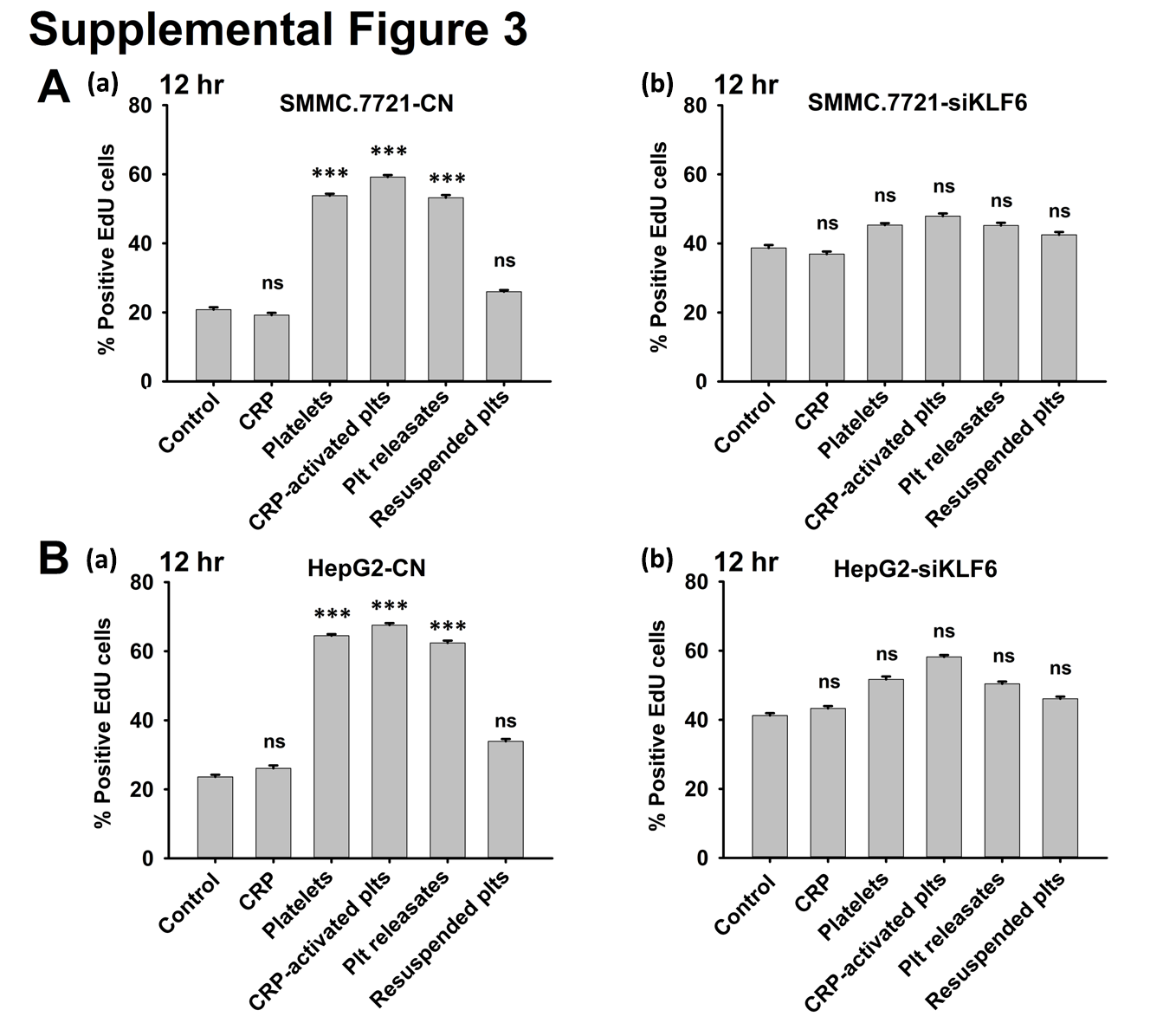
**

**Supplemental Figure 3. The down-regulation of KLF6 in HCC cells interrupted the proliferative effect of platelets.** SMMC.7721 (A) and HepG2 (B) cells transfected with control shRNA (a, CN) or KLF6 shRNA (b, siKLF6) were incubated with platelets and their releasates. EdU incorporation was measured to assess HCC cell proliferation after incubation for 12 hours, and the respective cell types cultured in fresh medium served as a control. The data are presented as the mean ± SEM (***p<0.001 compared with controls. Five fields were randomly selected and examined at ×200 magnification, and the experiment was repeated three times.).

**
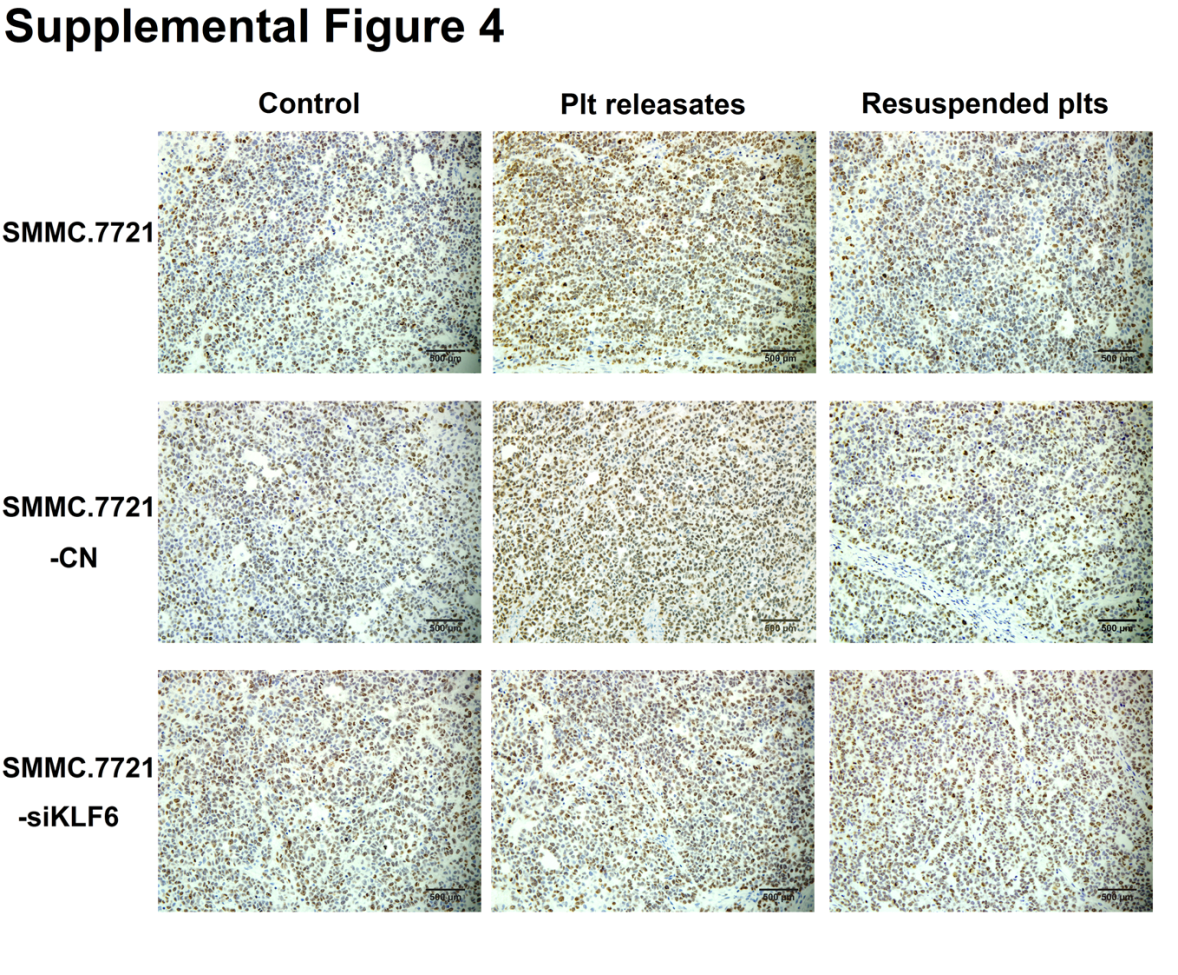
**

**Supplemental Figure 4. Platelet releasates increase Ki67 expression in SMMC.7721 subcutaneous tumors.** SMMC.7721 cells and shRNA-transfected SMMC.7721 cells were treated with platelet releasate and resuspended platelet pellets for 24 hours, and the tumor cells were then subcutaneously injected into the flanks of nude mice. Representative immunohistochemical staining for Ki67 in each group is shown.

**
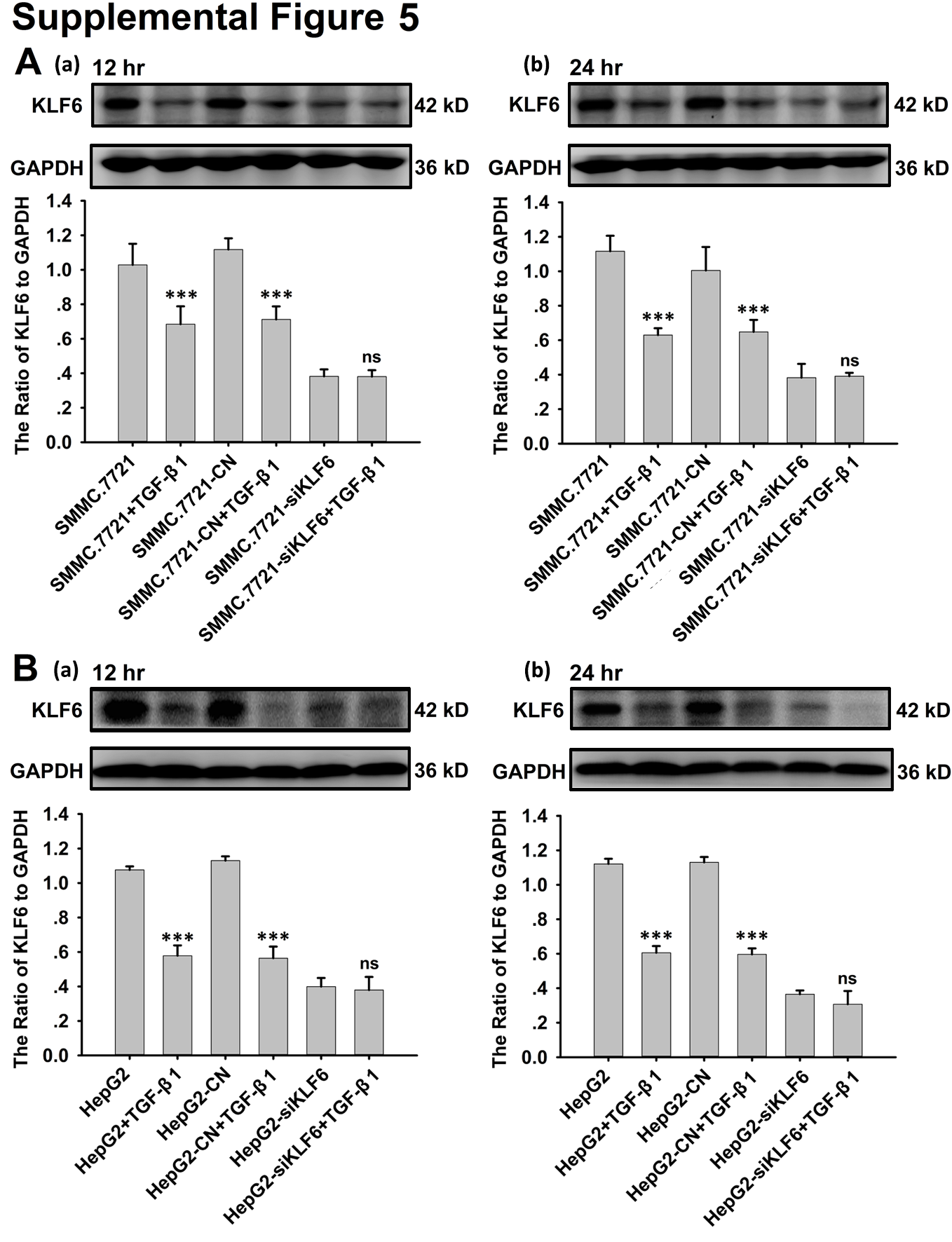
**

**Supplemental Figure 5. Recombinant TGF-β1 decreased expression of KLF6 in control HCC cells but not KLF6 silenced HCC cells.** SMMC.7721 (A) and HepG2 (B) cells transfected with control shRNA (CN) or KLF6 shRNA (siKLF6) were treated with human recombinant TGF-β1 (10 ng/ml) for 12 (a) or 24 (b) hours, expression of KLF6 was measured by a Western blot analysis. Cells cultured in fresh medium were used as a control. A quantitative analysis of the ratio of KLF6 to GAPDH expression is shown, and data are presented as the mean ± SEM (n = 3, ***p<0.001 compared with control).

**
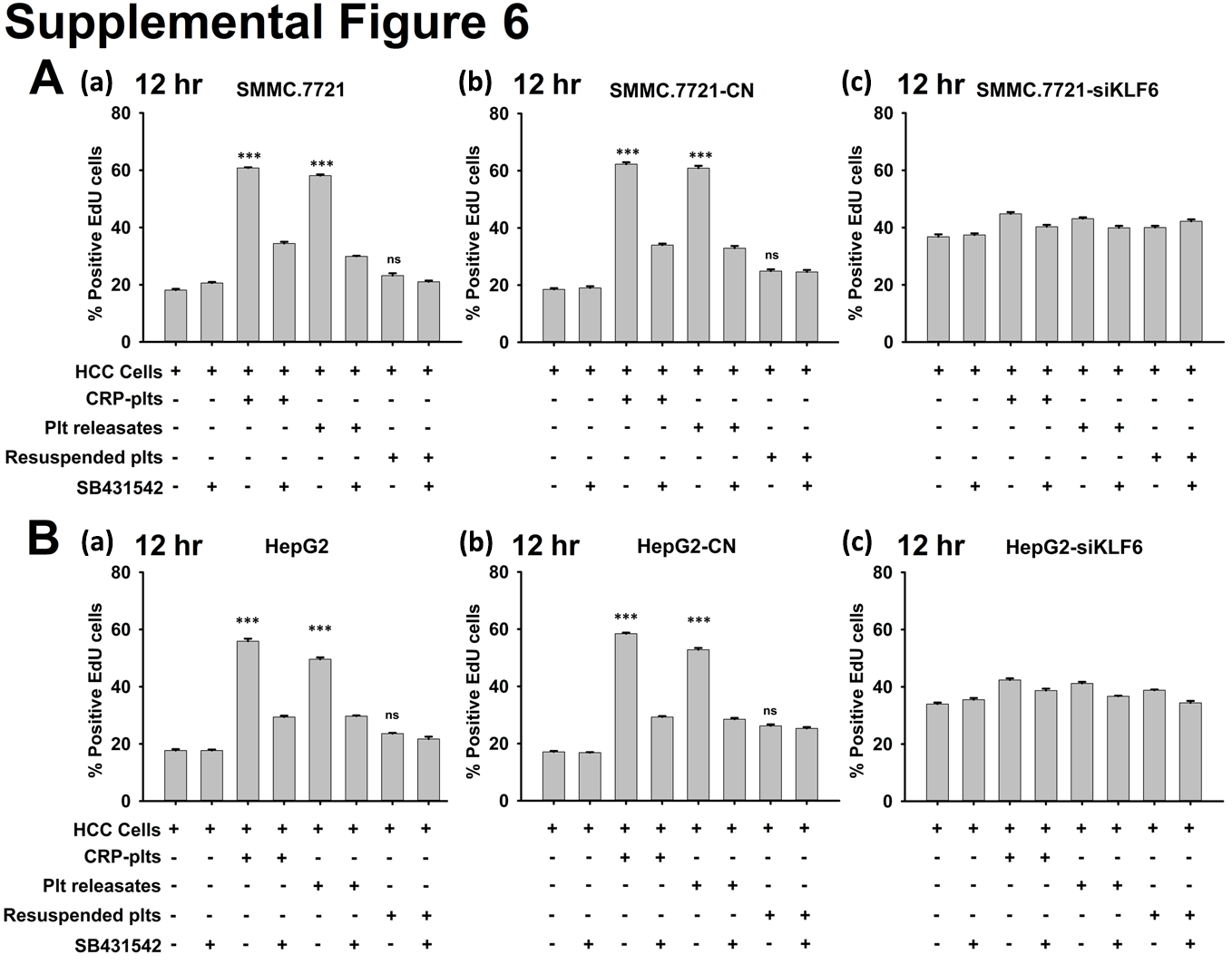
**

**Supplemental Figure 6. TGF-β inhibitor attenuated the promotion of HCC cell proliferation by platelets.** (A) SMMC.7721 (a), control shRNA-transfected SMMC.7721 (b) and KLF6-silenced SMMC.7721 (c) cells were treated with platelets and their releasates in the absence and presence of TGF-β receptor inhibitor SB431542 (10 μM) for 12 hours. Cell proliferation was assessed with the EdU incorporation assay. (B) Quantification of EdU-positive HepG2 (a), control shRNA-transfected HepG2 (b) and KLF6-silenced HepG2 (c) cells after treatment with platelets and their releasates in the absence and presence of SB431542 (10 μM) for 12 hours; cells cultured in fresh medium were used as a control. For (A) and (B), each experiment was repeated at least 3 times in triplicate, and 5 fields were randomly selected and examined at ×200 magnification for each group. Data are presented as the mean ± SEM. (**p<0.01, ***p<0.001 compared with controls).
